# Retroperitoneal laparoscopic partial nephrectomy with segmental renal artery clamping for cancer of the left upper calyx: a case report

**DOI:** 10.1186/s12894-017-0264-9

**Published:** 2017-08-31

**Authors:** Yajie Yu, Chao Liang, Meiling Bao, Pengfei Shao, Zengjun Wang

**Affiliations:** 1 Department of Urology, The First Affiliated Hospital of Nanjing Medical University, Nanjing, 210029 China; 2 Department of Pathology, The First Affiliated Hospital of Nanjing Medical University, Nanjing, 210029 China

**Keywords:** Laparoscopic partial nephrectomy, Retroperitoneal, Renal pelvis cancer, Segmental renal artery clamping, Upper calyx

## Abstract

**Background:**

Currently, the standard treatment for renal pelvis carcinoma is radical nephroureterectomy with bladder cuff excision. To describe the feasibility of retroperitoneal laparoscopic partial nephrectomy with segmental renal artery clamping for cancer of renal pelvis, we report this special case for the first time.

**Case presentation:**

A 67-year-old woman received this operation. Preoperative ureteroscopy revealed a papillary neoplasm with a pedicle in the upper calyx of the left kidney. After entering the retroperitoneal space and dissociating the renal artery and renal vein, the target artery was clamped beyond the final bifurcation before entering the parenchyma. After incision of the left renal parenchyma and exposure of the upper calyceal neck, the tumor was found confined to the upper calyx. Thereafter, the renal calyx and parenchyma were sutured successively after complete resection of the neoplasm. Postoperative pathological examination confirmed that the Grade I papillary carcinoma was confined to the mucosal layer. Thus far, there is no evidence of recurrence during the follow-up period for more than 42 months after surgery.

**Conclusions:**

Retroperitoneal laparoscopic partial nephrectomy with segmental renal artery clamping of the kidney provides a feasible treatment modality for noninvasive tumors that are limited to the calyx.

## Background

Upper tract urothelial carcinomas (UTUCs) are defined as tumors arising anywhere along the urothelial lining of the urinary tract from the renal calyces to the ureteral orifice. Currently, the standard treatment for renal pelvis carcinoma is radical nephroureterectomy with bladder cuff excision. In the current case, considering the fact that the patient had bilateral renal insufficiency and the tumor was localized, laparoscopic partial nephrectomy (LPN) with segmental renal artery clamping was eventually performed.

## Case presentation

**Fig. 1:**
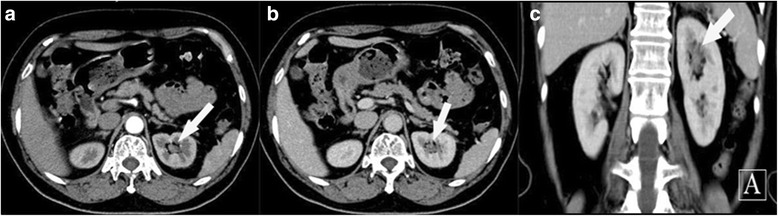
Enhanced abdominal computed tomography revealing a soft tissue density (*white arrow*) in the upper calyx of the left kidney (**a**) Cross section, arterial phase (**b**) Cross section, venous phase (**c**) Coronal plane

**Fig. 2:**
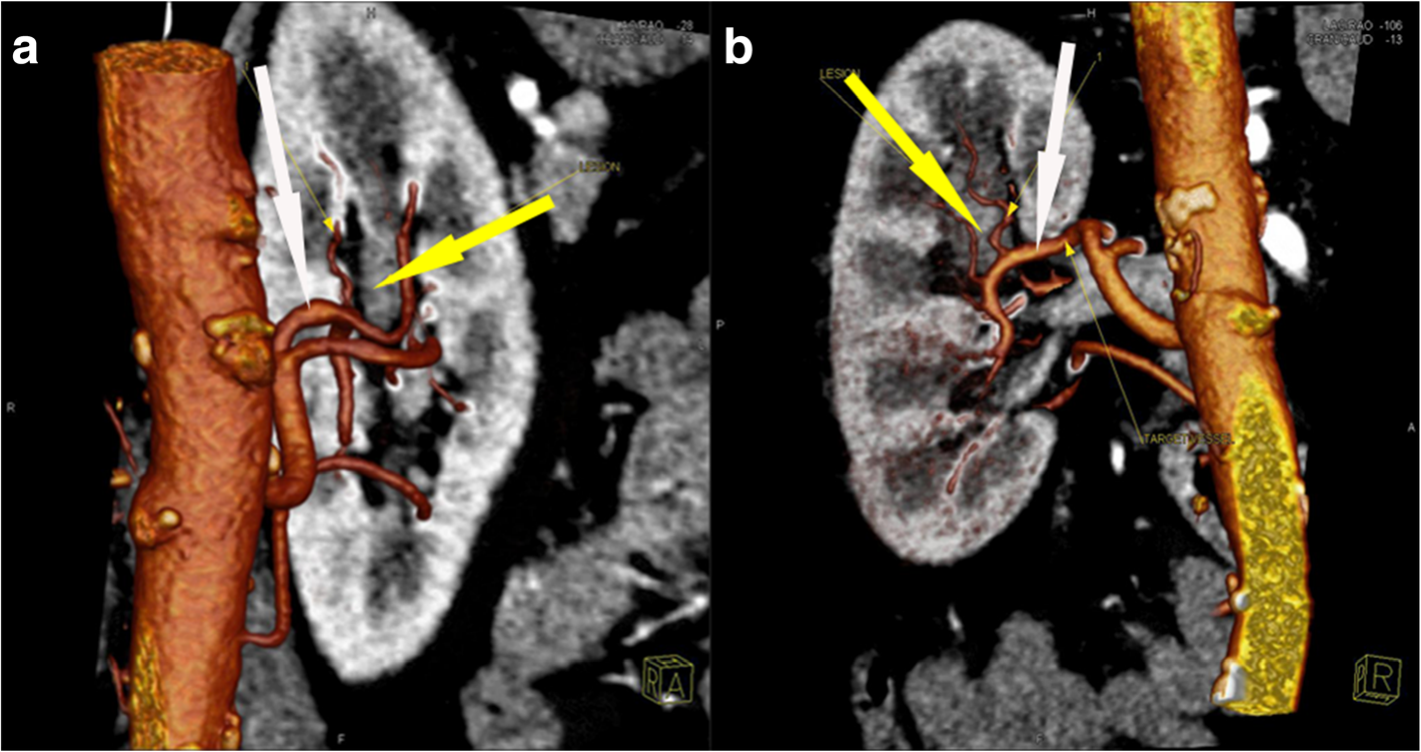
Computed tomography angiography showing the blood supply vessels (*white arrow*) of tumor region (*yellow arrow*) (**a**) Anterior view (**b**) Right view

A 67-year-old woman who complained of gross hematuria for 3 days was admitted to our department on November 21, 2012. The patient reported hematuria without fever, and denied having low back pain or edema of lower limbs. She had been diagnosed with diabetes, but was not undergoing regular treatment. Her main laboratory examination and ancillary investigation results were as follows. Routine urine test showed occult blood in the urine, with ++ cells/μL. Biochemical examination showed a blood sugar level of 8.36 mmol/L. The serum creatinine (SCr) level and estimated glomerular filtration rate (eGFR) were 93.5 μmol/L and 60.78 ml/min/1.73 m^2^, respectively. Urine cytology test showed the presence of tumor cells twice. Enhanced abdominal computed tomography (CT) revealed a soft tissue density in the upper calyx of the left kidney, suggesting pyelo-carcinoma (Fig. 1). Computed tomography angiography (CTA) identified the blood vessels that supply the tumor region (Fig. 2).

**Fig. 3:**
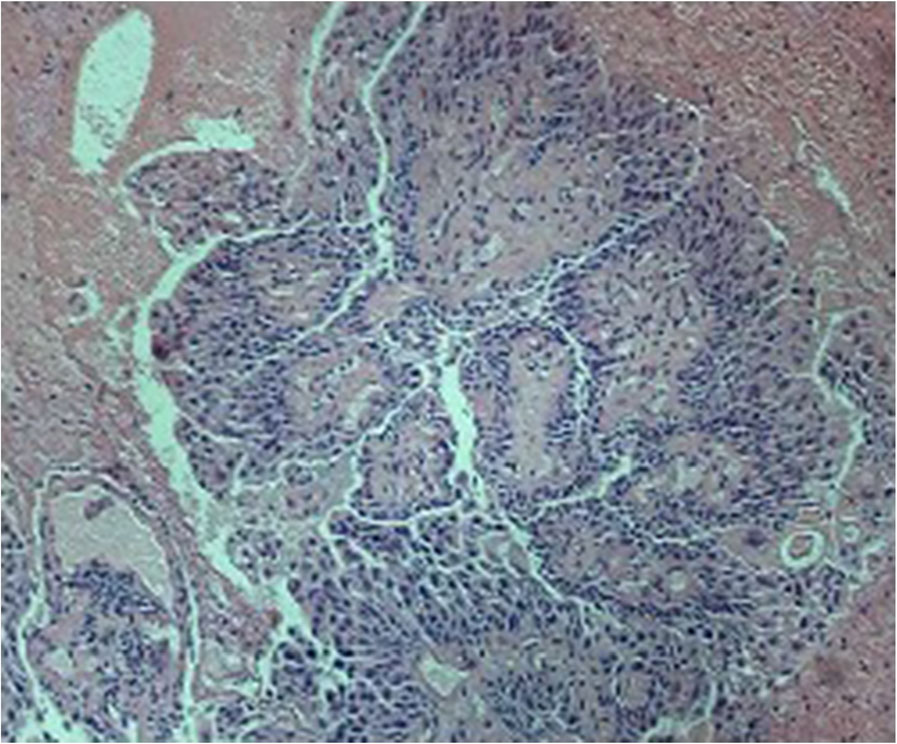
Histopathologic examination of the surgical specimens revealing grade I papillary carcinoma of the renal pelvis

After admission, the patient received aggressive perioperative treatment in order to improve her cardiac and renal function, and to control her blood sugar levels, so as to improve her tolerance to surgery. Ureteroscopy detected a papillary neoplasm with a pedicle in the upper calyx of the left kidney. Subsequently, the patient underwent LPN with segmental renal artery clamping. After entering the retroperitoneal space and dissociating the renal artery and renal vein, the target artery was clamped beyond the final bifurcation before entering the parenchyma. Access and clamping strategies for target arteries were preoperatively determined on the basis of the 3D models by the radiologist and surgeon. After incision of the left renal parenchyma and exposure of the upper calyceal neck, the tumor was found confined to the upper calyx. Later, the renal calyx and parenchyma were sutured successively after complete resection of the neoplasm. During the operation, a double J tube was placed for drainage of urine and for postoperative infusion therapy. The tumor was found to be confined to the upper calyx of the left kidney. Histopathological examination of the surgical specimens confirmed a diagnosis of papillary Grade I carcinoma of the renal pelvis, with massive hemorrhage, with the tumor measuring 18 mm × 10 mm × 7 mm and confined to the mucosal layer (Fig. 3). All surgical margins were negative.

The patient received postoperative intravesical instillation of pharmorubicin fortnightly for 3 months. Subsequently, the double J tube was removed. A cystoscopy was performed every 3 months for the first 3 years. CT was performed to check for the recurrence or metastasis of the tumor every 6 months for the first 3 years, and annually thereafter. There was no evidence of recurrence during the follow-up period for more than 43 months after surgery till the time of writing this report. The most recent SCr level and eGFR of the patient were 82.9 μmol/L and 69.78 mL/min/1.73 m^2^, respectively.

## Discussion

Urothelial carcinomas (UCs) are the fourth most common tumors after prostate (or breast), lung, and colorectal cancer [1]. Bladder tumors are the most common malignancy of the urinary tract and account for 90%–95% of the UCs [2]. In contrast, UTUCs are uncommon and account for only 5%–10% of the UCs [3]. Renal pelvic carcinoma accounts for the vast majority of UTUCs. In more than 95% of the cases, renal pelvic carcinoma is derived from the urothelium [4]. The WHO classification of bladder cancer in 1973 that distinguished it into three grades (G1, G2, and G3) is the most widely used method of tumor classification. The most common symptom of pyelo-carcinoma is gross or microscopic hematuria, observed in 70%–80% of the cases [5], followed by flank pain and a lumbar mass. UTUCs that invade the muscle wall usually have a very poor prognosis. The 5-year specific survival is less than 50% for pT2/pT3 tumors and less than 10% for pT4 tumors [6].

Radical nephroureterectomy with excision of the bladder cuff is the gold standard treatment for renal pelvis carcinoma [7]. However, for the superficial isolated tumor localized to one particular calyx, the advantage of LPN with segmental renal artery clamping is more obvious in patients with renal insufficiency or in those with high risk factors. Compared with radical nephrectomy, our novel segmental clamping techniques block the feeding arteries to the tumor, thus avoiding whole renal ischemia and protecting residual renal function. Compared with endoscopic management, this technique has the advantages of more thorough excision and low tumor recurrence rate.

The indication for this operation is superficial low-grade cancer of the renal pelvis that is localized to one particular calyx.

This technique (retroperitoneal laparoscopic partial nephrectomy with segmental renal artery clamping) was pioneered by our team and has been routinely carried out in our department [8]. After long-term promotion and verification, this technology has been proved to be safe and effective.

The key points of this operation were as follows. Firstly, the target artery was determined preoperatively by building a CTA model [8]. Secondly, the renal artery was blocked selectively to protect renal function. Thirdly, after clamping the target artery, the renal parenchyma was incised to expose the upper calyceal neck. A wedge resection was performed to ensure that the neoplasm had been resected in extenso. Lastly, a double J tube was placed during the operation to reduce the risk of postoperative leakage of urine and to facilitate postoperative perfusion treatment.

There do exist risk of tumor spillage and seeding. In this case, to prevent cancer spillage or seeding, we suggest guaranteeing the resection range be sufficient and avoiding touching and squeezing the tumor. When the tumor is cut off, immediately put it into a specimen bag and then suture the wound. At last, we could flush the surgical area with sterile water.

Until the time of writing this report, there was no evidence of recurrence during the follow-up period for more than 43 months after surgery, and the kidney function of the patient afforded her a normal daily life.

## Conclusions

In a word, for patients having superficial low-grade renal pelvic cancer localized to one particular calyx, retroperitoneal LPN with segmental renal artery clamping provides a new feasible strategy to ensure complete resection of the tumor, while simultaneously preserving normal renal function.

## Abbreviations

CT: Computed tomography
CTA: Computed tomography angiography
eGFR: estimated glomerular filtration rate
LPN: Laparoscopic partial nephrectomy
SCr: Serum creatinine
UCs: Urothelial carcinomas
UTUCs: Upper tract urothelial carcinomas
